# Topographic Orientation of Scaffolds for Tissue Regeneration: Recent Advances in Biomaterial Design and Applications

**DOI:** 10.3390/biomimetics7030131

**Published:** 2022-09-12

**Authors:** Jiayu Chi, Mingyue Wang, Jialin Chen, Lizhi Hu, Zhixuan Chen, Ludvig J. Backman, Wei Zhang

**Affiliations:** 1 School of Medicine, Southeast University, Nanjing 210009, China; 2 Jiangsu Key Laboratory for Biomaterials and Devices, Southeast University, Nanjing 210096, China; 3 China Orthopedic Regenerative Medicine Group (CORMed), Hangzhou 310058, China; 4 Department of Integrative Medical Biology, Anatomy, Umeå University, SE-901 87 Umeå, Sweden; 5 Department of Community Medicine and Rehabilitation, Physiotherapy, Umeå University, SE-901 87 Umeå, Sweden

**Keywords:** biomaterial, biomimetics, orientation, scaffold, tissue engineering, tissue regeneration, topography

## Abstract

Tissue engineering to develop alternatives for the maintenance, restoration, or enhancement of injured tissues and organs is gaining more and more attention. In tissue engineering, the scaffold used is one of the most critical elements. Its characteristics are expected to mimic the native extracellular matrix and its unique topographical structures. Recently, the topographies of scaffolds have received increasing attention, not least because different topographies, such as aligned and random, have different repair effects on various tissues. In this review, we have focused on various technologies (electrospinning, directional freeze-drying, magnetic freeze-casting, etching, and 3-D printing) to fabricate scaffolds with different topographic orientations, as well as discussed the physicochemical (mechanical properties, porosity, hydrophilicity, and degradation) and biological properties (morphology, distribution, adhesion, proliferation, and migration) of different topographies. Subsequently, we have compiled the effect of scaffold orientation on the regeneration of vessels, skin, neural tissue, bone, articular cartilage, ligaments, tendons, cardiac tissue, corneas, skeletal muscle, and smooth muscle. The compiled information in this review will facilitate the future development of optimal topographical scaffolds for the regeneration of certain tissues. In the majority of tissues, aligned scaffolds are more suitable than random scaffolds for tissue repair and regeneration. The underlying mechanism explaining the various effects of aligned and random orientation might be the differences in “contact guidance”, which stimulate certain biological responses in cells.

## 1. Introduction

Due to injury and aging, various tissues of the human body are affected by pathological defects. Clinical data show that most of the pathological tissues are unable to regenerate spontaneously or to a satisfactory degree with regained function. Due to the fact that a lack of donors limits the possibility of performing traditional surgery, and that the recipient’s immune system affects the outcome, alternative approaches, including tissue engineering, have attracted much attention [1]. The purpose of tissue engineering is to develop alternatives for the maintenance, restoration, or enhancement of injured tissues and organs [2,3]. The three elements of tissue engineering refer to cells, scaffolds, and growth factors. Among these three elements, scaffolds play a significant role, as they provide an artificial microenvironment for cell growth. Recent advances in tissue engineering have focused on the development of biomimetic scaffolds that simulate the extracellular matrix (ECM) of native tissues regarding both structure and composition [4,5]. 

The ECM is an important component present within all tissues and organs, and provides not only essential physical scaffolding for the cellular constituents but also initiates crucial intercellular communication. It has been identified that native ECM has unique structures and differs between tissues according to their function [5]. For example, ECM in specific tissues, such as nerves, muscles, and tendons, exhibits a unique geometrical organization closely related to their respective functions, namely directional signal transfer, global contraction, and longitudinal mechanical toughness, respectively [6,7]. 

The topographical properties, including the patterns, roughness, and porosity of the scaffolds are important for the regulation of physiologically relevant cellular functions [8]. The topographic orientation of ECM can be roughly divided into the following two categories: aligned and random. It has been reported that various living cells exhibit high sensitivity to the topographic orientation of scaffolds both in vivo and in vitro [6,8,9,10]. For example, it is shown that the topographic orientation of native ECM significantly affects cell behaviors, including morphology [11], migration [12], proliferation [13], and differentiation [14]. In addition to the topographic orientation, the roughness and porosity of scaffolds also play a crucial role in the regulation of cell behaviors [8]. Thus, in order to more sufficiently achieve the desired effects, the topographical features of the scaffolds for tissue engineering should be tailored specifically for each tissue to highly mimic ECM with particular characteristics [15]. For example, Akbar et al. designed a functionally graded scaffold for mimicking cancellous bone architecture, and demonstrated excellent permeability, making it ideal for the repair of cancellous bones [16].

In the past, most reviews focused on the effect of scaffold topography on single tissues (e.g., bone [17,18], nerve [8,19,20], skeletal muscle [21], etc.), and the effect of the morphological characteristics of scaffolds made by different materials (e.g., hydrogels [22], graphene [23], etc.) or fabrication methods (e.g., electrospinning [24], etching [25], etc.) for tissue regeneration. Although the review by Lu’s specifically described the application of aligned scaffolds in tissue engineering, we have not found a review in the past five years that has compared the advantages and disadvantages of different scaffold topographic orientations, nor their underlying mechanism in tissue regeneration [26]. A review by Wang analyzed the applications of aligned scaffolds in some tissues under the section of fabrication methods, but the biological properties of scaffolds with different topographic orientations was not elucidated [27]. Thus, a review that specifically compares the application of scaffolds with different topographic orientations in various tissues is still lacking.

This review focuses on recent studies based on scaffold topographic orientation for tissue regeneration and evaluates its prospects for future biomaterial design and tissue engineering applications. A brief overview of the various fabrication methods, followed by a comparison of the physicochemical and biological properties of scaffolds with different topographic orientations, are summarized. Applications of scaffold topographic orientation for various types of tissue regeneration, as well as their advantages and limitations, are discussed. 

## 2. Fabrication of Scaffolds with Different Topographic Orientations

### 2.1. Electrospinning

**Figure 1:**
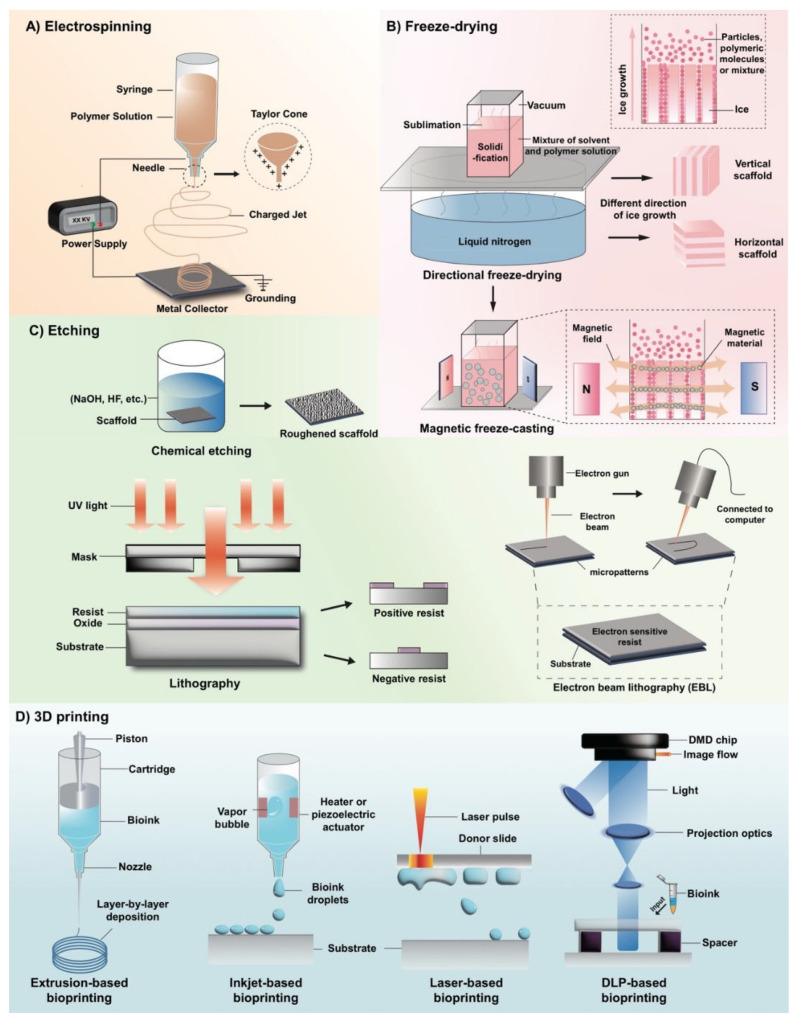
Schematic illustration of the fabrication of scaffolds with different topographies. (**A**) Electrospinning; (**B**) directional freeze-drying and magnetic freeze-casting; (**C**) etching; (**D**) 3D printing.

Thanks to the similarities between the resulting fibers in electrospinning and natural tissues, the technique has been widely applied in biomedical fields, such as tissue regeneration and drug delivery. We will briefly introduce the principles, advantages, and several applications of electrospinning in biomedicine. As shown in Figure 1A, the basic electrospinning device consists of a high-voltage power supply, a solution container with a nozzle, and a grounded metal collector. The positive electrode of the power supply is placed in the solution container, while the negative electrode is connected to the metal collector. In the process of electrospinning, a high electrostatic force is used to generate a charged jet from the polymer solution, and the continuous filaments are extracted or ejected through a nozzle [28]. Specifically, the high-voltage electrostatic field causes the charged polymer solution to be extruded from the nozzle. The surface tension of the solution and the force generated by the surface charge is opposite [29]. As the intensity of the electric field increases, the two forces interact and the hemispherical surface of the fluid gradually elongates, becoming a cone-shaped droplet—the Taylor cone [30]. When the electric field force is strong enough, the repulsive electrostatic force generated by the surface charge, the contraction force to the counter electrode can overcome the surface tension of the fluid, and the charged solution jet is ejected from the tip of the Taylor cone [31]. Due to flow instability and electrically driven bending instability, the charged polymer jet undergoes an unstable process after uniform extension, and the polymer is highly stretched. At the same time, due to the rapid volatilization of the solvent, nanoscale polymer materials are obtained on the collector [32,33].

The process of electrospinning is affected by several factors. The formation of electrospun fibers can be altered by changing the properties of the polymer solution (molecular weight, concentration, conductivity, polarity, surface tension, etc.), technical parameters (applied voltage, working distance from syringe to collector, speed of replenishing solution, the movement state of the collecting plate, etc.) and environmental factors (humidity, temperature, air velocity, etc.) [34,35]. This means that physical and chemical properties, such as material characterization, fiber diameter, conductivity, and porosity can be controlled by changing these parameters. Among the mentioned parameters, it is known that the movement state of the collector plate decides if the nanofibers are organized in an aligned arrangement or randomly. Hossain et al. used a conventional rotating drum approach to prepare PAN membranes, and they set the parameters to be either 300 rpm or 1300 rpm combined with 13–14 kV, 1.4 mL/h, and 15 cm, accordingly. They found that fibers produced with the lower speed of 300 rpm resulted in random organization to a higher extent as compared to the higher rotation speed of 1300 pm [36]. Courtney et al. produced PFUU fibers by applying electrospinning at varying velocities ranging from 0 to 13.8 m/s. They found that a velocity of 3.0 m/s or more resulted in aligned fiber network, i.e., alignment increased with increasing speed [37]. Taken together, a random scaffold can be obtained when the collector is stationary or rotating at a low speed, while an aligned scaffold will be obtained at a higher rotating speed.

Electrospinning is recognized as one of the most useful techniques to fabricate topographical features in tissue engineering. It is a simple method to use for the fabrication of uniform scaffolds with desired features, such as orientation, porosity, mechanical properties, and with a structure similar to that of ECM in natural tissues [34]. However, there are still some shortcomings, such as the use of a toxic solvent, that they are difficult to transport and store, and the insolubility of the polymer. Recently, the emergence of strategies, such as co-electrospinning using blends of solutions, has provided prospects for advances in electrospinning technology [38]. 

### 2.2. Freeze-Drying

Freeze-drying is a technology widely used in tissue engineering, biopharmaceuticals, food processing, and other fields. In recent years, it has been extensively studied as a method for fabricating novel porous scaffolds in tissue engineering [39]. As shown in Figure 1B, the manufacturing of scaffolds using freeze-drying is based on the solidification and sublimation of solvents [26,40]. When producing a scaffold using the freeze-drying method, a certain polymer of a desired concentration is dissolved in the solvent and, subsequently the mixture is frozen. By creating vacuum conditions, the solvent sublime and the remaining material have a fixed shape [39]. During this process, pore structure is obtained by replicating the structure of ice crystals [39], which means that the topographic feature of scaffolds prepared by freezing technology is fundamentally dependent of the morphology of the ice crystals. Thus, the topographic feature of scaffolds is influenced by many factors that have an impact on the growth and structure of ice crystals during freezing, including freezing temperature, solution concentration, solid content, polymer molecular weight, and the control of the freezing direction [39,41,42]. We will introduce several common methods that adjust temperature-related variables to design scaffolds with certain topographic orientations.

It has been reported that if a constant freezing temperature is used with the most traditional direct cooling and vacuum sublimation, scaffolds had random pores which can be called isotropic inner architecture [41]. However, different freezing temperatures influence pore size, as follows: a lower freezing temperature results in smaller pores, and a higher freezing temperature results in larger pores. This is due to the balance between the ice crystal nucleation rate and the ice crystal growth rate [39]. Supercooling is necessary for the nucleation of ice crystals, and the degree of supercooling directly affects the nucleation rate of ice crystals and, subsequently, the growth rate of ice crystals. In the high supercooling region, the ice crystal nucleation rate is higher than the ice crystal growth rate and, thus, scaffolds have more pores and a smaller pore microstructure. In contrast, in the low supercooling region, the nucleation rate of ice crystals is lower than the growth rate of ice crystals and, thus, scaffolds have larger pores in the final material [39].

By controlling the direction of coagulation and the growth direction of ice crystals (a process called “directional freezing”), scaffolds with aligned porous or channeled structures can be obtained [26,39,41,43]. It has been widely reported that directional freezing could be achieved by choosing the appropriate cooling rate or by producing a temperature gradient between the two sides of freezing samples [39,41,44]. The principle is that the solvent coagulates from one side to the other and the ice crystals are formed in one direction. The solution or particles used are concentrated and excluded between the growing ice crystals. Finally, the aligned structures are obtained following the removal of the orientated ice by freeze-drying [44]. 

Although freeze-drying has become one of the most promising techniques for the fabrication of porous scaffolds [45], the aligned scaffolds manufactured by the traditional directional freeze-drying technique only exhibit excellent mechanical properties in the direction parallel to the freezing direction. In the direction vertical to the freezing direction the scaffolds are fragile [46], which limits the use of such scaffolds in tissues that require multidirectional forces, such as bone [47]. To solve this problem, magnetic freeze-casting emerges as an improvement on the traditional directional freeze-drying technique by exerting the magnetic field force to produce dedicated aligned scaffolds with increased strength and stiffness. As shown in Figure 1B, the core of the magnetic freeze-casting method is the interaction of the magnetic material in the casting solutions with an external magnetic field [47]. By adjusting the magnetic field to be oriented radially, axially, or transversely to the freezing direction, different topographic structures can be obtained [47]. When a transverse magnetic field is applied, the magnetic material in the casting solutions is arranged according to the magnetic field, forming aligned pore channels vertical to the ice growth direction. The following two modes of microstructural alignment can be observed: the lamellar walls which are parallel to the growing ice crystals, and the mineral bridges which are aligned according to the magnetic fields. Scaffolds with this microstructure have enhanced mechanical strength in multiple directions, which is suitable for bone generation [26,47,48]. Additionally, “core-shell” structure and “core-shell” gradient architecture with dense outer perimeters surrounding porous inner cores can be obtained by the radial and axial fields [26,48]. 

Recently, progression has been made in the research of magnetic freeze-casting. Frank et al. successfully constructed aligned scaffolds with magnetized alumina particles [49,50]. Furthermore, it has been proposed that a single even triaxial nested Helmholtz coil could be substituted for permanent magnets for a more uniform magnetic field, which could prevent particle agglomeration and reduce the unintended stress concentration in scaffolds [51,52]. In summary, magnetic freeze-casting may enable the development of scaffolds with multidirectional mechanical strength which can mimic the structure of natural tissues, such as bone.

In general, freeze-drying technology has many advantages, such as high porosity and interconnectivity, as well as outstanding biocompatibility [40]. The freeze-drying process also avoids using toxic solvents. In addition, the low temperature in the process prevents the deactivation of biological macromolecules (e.g., proteins and enzymes) and drugs [39,40]. Therefore, this method has unique advantages in aqueous systems. However, scaffolds produced by the traditional freeze-drying still have a few limitations, such as insufficient inherent strength [26]. To obtain layered or 3D structures and to manufacture scaffolds with better properties, this technology has also continued to innovate and develop. In addition to magnetic freeze-casting [47], many new methods, such as bidirectional freezing [47] and electric field freeze-drying, have emerged [53].

### 2.3. Etching

Lithography, electron beam lithography (EBL), and chemical etching are commonly used to fabricate tissue engineering scaffolds (Figure 1C) [54]. Among these approaches, only lithography and EBL can manufacture scaffolds with aligned topographies. In lithography, a photomask with a designed pattern is positioned on a substrate full of photoresists before being exposed to ultraviolet (UV) light that selectively irradiates the photoresist in the area not covered by the photomask. In the developer, the crosslinked area is insoluble and the non-crosslinked area is soluble and washed away, thus, enabling a transfer of patterns [25,26,54,55,56]. The resolution of lithography is about 1–2 μm [54]. The process of EBL is similar to lithography. However, instead of UV light, a high-energy electron beam emitted by an electron source is used, and the photoresist on the substrate is replaced with an electron-sensitive resist. Instead of a mask, it uses a computer to control the exposure of the electron beam on the resist, so that degradation or cross-linking interactions occur, fabricating a specific stereoscopic image [25,54,57]. The EBL method can achieve a resolution below 10 nm [58]; however, it takes a long time to expose [54]. In recent years, there have been some improvements in the process. Jumbertde et al. used electron beam exposure to strengthen the adhesion between MoS2 and PDMS for direct lithography, simplifying the original production process. At the same time, because no electron-sensitive resist was used, there would be no polymer residues in the sample [59]. Ice lithography (working at low temperature with vapor-deposited organic molecules used as electron-sensitive photoresists) as an advanced stage of EBL allows etching on substrates of any shape and size, and because the meteorological precipitation of ice is used as an electron-sensitive resist, the manufacturing process is more conducive to the environment [60]. 

The advantage of designed patterns is that they can form a specific nano-microenvironment to regulate the growth and differentiation of cells. For instance, nano-grooved scaffolds contribute to the extension of nerve cells [46,56]. Meanwhile, using lithography to manufacture a PLGA scaffold with specific nano-topography results in highly stretched cells, promoting differentiation into cardiomyocyte lineages [57]. However, the expensive equipment and high environmental cleanliness requirements for fabricating aligned patterns prevent widespread use at present [54].

Unlike aligned micropatterns, the random patterns are disordered in orientation and organization. The random patterns are mostly formed spontaneously, and often only superficially, with uneven and rough surfaces. Such patterns are mainly fabricated by chemical etching, which is basically a kind of surface modification [25]. Using chemical etching, materials are soaked in chemical corrosives (such as hydrofluoric acid and sodium hydroxide) to obtain nanoscale landforms and rough surfaces [25]. This method is a simple, cheap, and flexible way to create nanostructures on the surface of materials and, thus, is capable of adjusting the growth parameters of interest to specific cells, such as osteoblasts. Additionally, this method allows for the formation of polar groups, making the material more bioactive [61]. In a study by Curtis et al. it was found that the adhesive ability of cells in an aligned topography was even lower than that of a flat surface. They also found that the rough surface in the random topography could have a positive effect on cell adhesion within a certain range [57,62]. The aligned and random topographies have their own advantages and disadvantages. The specific choice depends on the natural properties of the tissues to be repaired.

### 2.4. 3D Printing

The 3D printing method has been applied in various fields as a new technology to fabricate desired architectures of scaffolds [63]. By using CAD software to produce the architecture, various scaffolds with aligned, random, or more complicated topographic orientations can be obtained [40,64]. The 3D printing method is regarded as a transformative technology with various advantages, such as greater flexibility, higher efficiency, and lower cost [63]. We will explain the following three currently prevailing 3D bioprinting techniques: inkjet-based bioprinting, extrusion based bioprinting, and light-assisted bioprinting (as shown in Figure 1D).

Similar to traditional jetting printing, the difference between inkjet-based bioprinting and the traditional method is that it distributes protein or cell solutions drop by drop rather than ink [65]. To assemble a printed 2D layered structure into a 3D structure, adhesives, such as thermosensitive gels, have been used by researchers to create a sophisticated biological scaffold [66]. Although inkjet-based bioprinting has advantages, such as low cost and hydrated products [65], it is still considered a questionable technique due to its low droplet directionality, nozzle clogging, the risk of exposing cells to thermal and mechanical stress, and the difficulty of integrity, etc [63].

Extrusion-based bioprinting releases biomaterial more continuously as compared to inkjet-based bioprinting [67]. It is based on a robotic solid freeform fabrication platform with a gel deposition instrument that deposits materials layer by layer to form a 3D scaffold [67]. The biological 3D printer involves the main movement of the X, Y, and Z axes by the relative movement of the deposition table and the printing nozzle. Researchers have used multicellular spheroids as building blocks to manufacture organs successfully [68]. With the application of new biomaterials, various biological products can be obtained, such as ECM, liver, cartilage, ear, etc. [69,70]. The technology is simple, flexible and inexpensive. However, it is associated with disadvantages, as it may cause damage to cells, integrity issues, etc. [63].

Light-assisted bioprinting differs from how traditional bioprinters work and offers great advantages by utilizing the interaction between light- and photo-polymerization to construct sophisticated 3D scaffolds [71,72]. It includes the following two groups: DLP-based printers and laser-based printers [63]. The resolution of light-assisted bioprinting is dependent on the focal size of the light beam from each micromirror and the wavelength of the laser. Since the focal size of the light beam is at micron scale, the technique is very accurate [73,74]. In addition, it has further advantages, such as high efficiency and high biocompatibility. However, some drawbacks exist, such as the limitations of photosensitive materials as raw materials for manufacturing and the need for a larger storage space to host the raw materials [71,72].

Overall, as 3D printing technology develops, it attracts much attention in the field of bioengineering. Daly et al. designed a 3D printed cartilage scaffold with microchannels that can promote angiogenesis to optimize the strategy of tissue engineering to repair cartilage [75]. Hong et al. used nano-clay material to 3D print highly stretchable tough hydrogel which is shown to be suitable for long-term cell cultures [76]. Kim et al. found an excellent way of regenerating myoblasts by 3D printing a PCL/PVA mixed solution and subsequently removing the PVA and performing a collagen microfiber coating [77]. Nevertheless, a few shortcomings regarding 3D printing technology still exist. For better physical guidance to biomolecules, the resolution of bioprinters needs to be improved to the nanometer scale [63]. Furthermore, high expense, scarce raw materials, and limited output are also urgent problems to be solved. 

## 3. Physiochemical Properties of Scaffolds with Different Topographic Orientations

### 3.1. Mechanical Properties

The alignment of nanofibers in scaffolds has a great impact on the mechanical properties [78]. Previous studies have reported that aligned scaffolds prepared by electrostatic spinning had higher module values and breaking forces than random scaffolds, which indicates a better capacity to resist the external force of aligned scaffolds [79,80,81,82]. Xie et al. demonstrated higher toughness in aligned scaffolds as compared to the random (an aligned value of 142.5 ± 97.9 vs. a random value of 52.7 ± 24.2 MPa). Similar results were also observed for tensile modulus and ultimate stress [79]. Analogously, Yin et al. found that the Young’s modulus of an aligned PLLA scaffold (22.76 ± 5.63 MPa) was 36 times higher as compared to random scaffolds (0.63 ± 0.56 MPa) [80]. Moreover, Yuan et al. reported that the tensile strength in aligned scaffolds in the dominant direction had higher values as compared to the perpendicular direction [83]. In general, an aligned scaffold exhibits superior mechanical properties as compared to a random scaffold and, thus, it is likely that aligned scaffolds have a better ability to adapt to the high pressure and force environment in the body [80]. However, contrary to this, Dias et al. reported that for skin substitutes, the reached elongation before rupture was longer in the random scaffolds as compared to the aligned scaffolds [81,84], suggesting a better stability of scaffolds when nanofibers are randomly arranged. Subramanian et al. reported a significant increase in the tensile strength and Young’s modulus of random PLGA fibers as compared to the aligned PLGA nanofibers. However, the aligned PLGA scaffolds reached longer elongation, meaning that they have a higher flexibility and elasticity as compared to the random PLGA [85]. Due to the complexity, the exploration of further studies on the mechanical properties of scaffolds should be divided into different tissues, force directions, and materials.

### 3.2. Porosity

The porosity of scaffolds play a critical role in tissue engineering. High porosity is necessary for the distribution and interconnection of homogeneous cells, which is beneficial for nutrient and oxygen diffusion [86,87]. The pore size in scaffolds fabricated by electrospinning is dependent on the microfiber diameter and density [85].

The porosity in aligned scaffolds is slightly increased as compared to random scaffolds [88]. However, Meng et al. showed that scaffolds with aligned nanofibers fabricated by electrospinning exhibited lower porosity as compared to random nanofibers [81]. Subramanian et al. found that the pore size of the aligned PLGA fibers fabricated by electrospinning was 3.5 ± 1.1 µm, which was significantly lower than that of the random ones (8.0 ± 2.0 µm); however, the porosity was comparable [85]. In conclusion, the topographic orientation is not decisive for the porosity.

### 3.3. Hydrophilicity 

Hydrophilicity refers to the physical property of materials that can form short-lived bonds with water through hydrogen bonds. It is an important property of scaffolds that is usually associated with enhanced biological behavior including cell attachment, viability, and proliferation [81,89,90]. It can be measured by the water-in-air contact angle—a material is hydrophilic when the contact angle of static water on the material surface is less than 90 degrees. Additionally, when the angle is smaller, the material is more hydrophilic [89,91]. The hydrophilicity of the material can be affected by many factors, such as the material itself and environmental conditions, as well as the structure of the material, such as the topographic orientation [90]. 

Firstly, scaffolds with coarser surfaces have better hydrophilicity because of the larger contact area between the rough nanofibers and water molecules. Furthermore, scaffolds with smaller fiber diameters can absorb more water due to the capillary effect [92]. It has been reported that poly(lactic-co-glycolic acid) (PLGA)/gelatin scaffolds with random orientation had a higher swelling ratio and hydrophilicity than aligned scaffolds [81]. This may be because the randomly oriented scaffolds manufactured by electrospinning have higher porosity, which results in better water adsorption due to the capillary effect [92]. However, Karimi et al. found significantly higher hydrophilicity in aligned poly(hydroxybutyrate)/chitosan scaffolds as compared to random scaffolds, while the pore diameters of the two scaffolds were similar [93]. Kai et al. reported that the effect of topographic orientation on the hydrophilicity was opposite on the poly(ε-caprolactone)/gelatin (PG) and poly(ε-caprolactone) (PCL) scaffolds [94]. 

The hydrophilicity of a scaffold is the result of surface smoothness, fiber diameter, pore size, etc [89,90]. The effect of these variables is known, but the effect of topographic orientation of scaffolds on hydrophilicity is still uncertain, and also depends on the materials used. Thus, when discussing the impact of the topographic orientation of scaffolds on hydrophilicity, it should be analyzed on a case-by-case basis.

### 3.4. Degradation

Degradation is of great importance in tissue engineering, since an ideal scaffold should make a balance between its degradation rate and the growth rate of neo-tissues [95]. It has been reported that aligned scaffolds possess a relatively slower degradation rate. Subramanian et al. reported that the percentage of molecular weight loss in a random PLGA scaffold fabricated by electrospinning was higher as compared to an aligned scaffold after 5 weeks in vitro, which shows that the aligned scaffolds possess prolonged degradation behavior [85]. Eslamian et al. demonstrated that scaffolds with highly aligned electrospun fibers had less burst release and more sustained release as compared to the random scaffolds which may be related to the relatively anisotropic degradation of aligned scaffolds [96]. Karimi et al. found similar results, i.e., that aligned poly-β-hydroxybutyrate (PHB)/chitosan fibrous scaffolds fabricated by electrospinning had slower degradation rates as compared to random scaffolds [97]. The reasons behind this phenomenon might be the lower rate of liquid diffusion in scaffolds with an aligned topography and, thus, the slower degradation rate [98,99,100]. The slow degradation rate of an aligned scaffold makes them promising for applications in the tissue engineering of tissues with slow regeneration, such as neural and bone tissue.

## 4. Biological Properties of Scaffolds with Different Topographic Orientations

### 4.1. Morphology and Distribution of Cells

**Figure 2:**
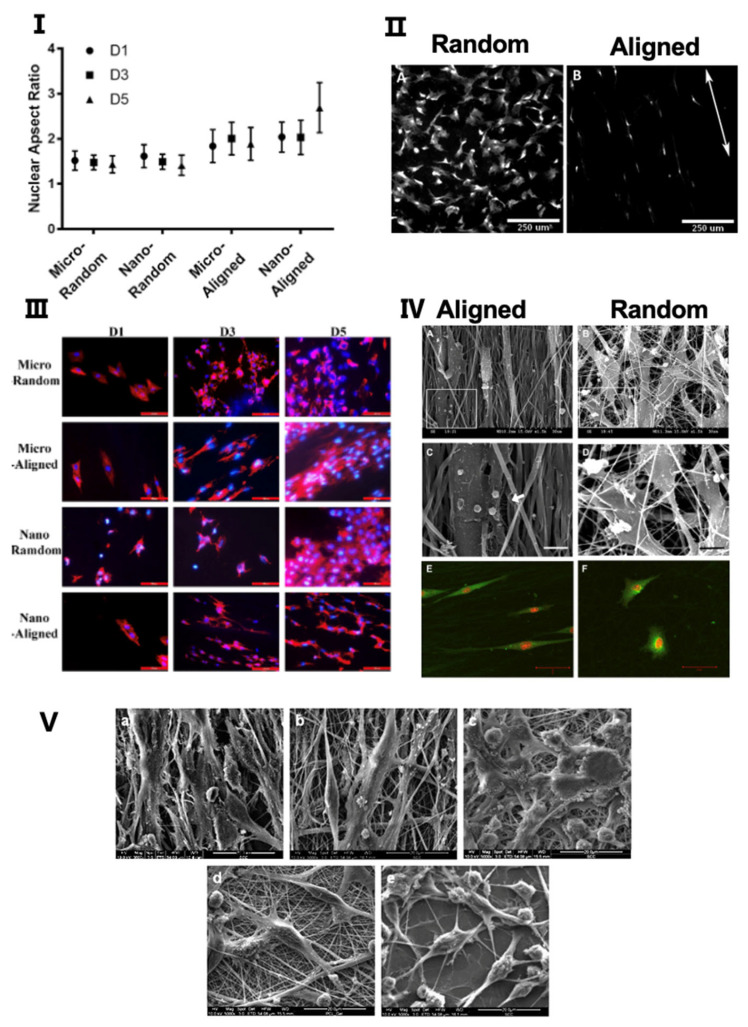
(**I**) Cell nuclei on aligned scaffolds had a higher aspect ratio as compared to nuclei on random scaffolds (Reproduced from [102], Elsevier Ltd., Amsterdam, The Netherlands). (**Ⅱ**) GFP-expressing rat dermal fibroblasts cultured on random collagen scaffolds exhibited random distribution, while cells seeded on aligned scaffolds exhibited elongated shape and were aligned in the direction of the long axis of fibers (arrows indicate the orientation of the aligned scaffolds) (Reproduced from [104], American Chemical Society). (**Ⅲ**) Cytoskeleton of HUVECs on different fibrous membranes on days 1, 3, and 5. Cytoskeletal F-actin (red) was shown at the cell periphery on randomly oriented nanoscale and microscale scaffolds, while cytoskeletal F-actin of cells were oriented parallel to the aligned nanoscale and microscale scaffolds (Reproduced from [102], Elsevier Ltd.). (**IV**) Morphological changes of tendon stem/progenitor cells grown on scaffolds. Here, B and F showed that cells on random scaffolds exhibited a stellate-patterned phenotype with randomly distributed features, while cells on aligned nanofibers (A,E) exhibited a classic fibroblast phenotype and are parallel to the fiber axis (Reproduced from [80], Elsevier Ltd.). (**V**) The SEM micrograph of Schwann cells on nanofiber scaffolds obtained after day 12 of cell culture. (**a**,**b**) showed that Schwann cells on oriented scaffolds were oriented along the fiber direction and aggregated around aligned fibers in a longitudinal manner, while (**c**,**d**) showed that cells grew in different directions on random fibers (Reproduced from [103], Elsevier Ltd.).

As shown in Figure 2, the topographic orientation of scaffolds significantly influences the growth characteristics of cells, especially the morphology and distribution of cells [101]. The morphology of cells, including the aspect ratio of cells, the aspect ratio of the nucleus, etc., is related to the biological activity of cells, such as proliferation, differentiation, etc. The distribution of cells is closely related to the mechanical strength and functional status of repaired tissue [102].

It is known that cells tend to align along microgrooves or similar topographic features on scaffold surfaces [101]. For scaffolds manufactured by electrospinning, cells seeded on random nanofibers exhibit irregular shapes and a random distribution, while cells seeded on the aligned scaffolds have elongated shapes and are aligned in the direction of the long axis of fibers [79,80,81,102]. Furthermore, the nuclei of cells on aligned scaffolds have a higher aspect ratio as compared to the nuclei of cells on random scaffolds [102]. It has also been reported that the arrangement of ECM is affected by the topographic orientations of scaffolds. Indeed, ECM-like collagen fibers exhibit a high degree of organization on aligned scaffolds, while they are arranged randomly on random scaffolds [79,80,101]. The effects of scaffold topographies on the morphology and distribution of cells have been validated on a variety of cells, such as fibroblasts [79], osteoblasts [81], endothelial cells [102], Schwann cells [103], etc. These cellular behaviors apply similarly to scaffolds fabricated by freeze-drying [104,105].

So far, the mechanism of how the topography of scaffolds affects cell bioactivities is still not elucidated. The current view is that cells can detect the biophysical properties of the underlying substrate, a phenomenon known as contact guidance, established by Weiss et al. in 1934 [106]. The cytoskeleton and the nucleoskeleton in the nucleus will undergo corresponding arrangement changes, which is why cells and nuclei exhibit different morphology on scaffolds with different topographies. This process facilitates the initiation of the translation of extrinsic mechanical signals into intracellular signals, as groups of cytoskeleton-associated molecules have been identified as potential upstream effectors of such substrate-induced signaling. As the intracellular signaling changes, both the transcriptional activities in the nucleus and the biological activity throughout the cell is altered [107,108,109,110]. This is also the reason why different shapes of nuclei are often related to different cellular activities [102]. The process when cells sense and respond to the topographic features of the substrate is called mechanosensing [111]. The mechanosensing in cells is different in different tissues.

### 4.2. Adhesion and Proliferation

The ability of cells to adhere and proliferate is a prerequisite for their specific function [112]. It has been identified that murine calvarial preosteoblasts have a better adhesion ability on random scaffolds as compared to aligned scaffolds, which may be attributed to the rough surface of random scaffolds [81]. Nevertheless, radially aligned scaffolds, fabricated by electrospinning, stimulate superior adhesion of dural fibroblast cells as compared to scaffolds with random collagen nanofibers [101]. KÖSE et al. reported that both the adhesion and proliferation of human MSCs were superior on aligned scaffolds as compared to random scaffolds [113]. It has also been reported that cells on aligned scaffolds grew and proliferated faster, as well as showed higher viability, as compared to cells grown on random scaffolds. This is true for rat Schwann cells, murine preosteoblasts, human endothelium cells, and human adipose-derived mesenchymal stem cells [81,102,114,115]. However, it was found that proliferation of tendon fibroblasts was not affected by fiber orientation [79]. In general, the effects of scaffold topographic orientation on adhesion and proliferation of different cells are inconclusive. Both adhesion and proliferation of cells are affected by multiple factors including scaffold topographic orientation, but also cell types, scaffold materials, etc. The underlying mechanism might be related to different signaling pathways initiated in cells.

Multiple signal pathways are related to the adhesion and proliferation of cells. For example, in the repair of many tissues, the Rho–ROCK pathway can increase the proliferation of smooth muscle cells but decrease the proliferation of embryonic stem cells [116]. It is shown that both proliferation and fibroblast-like behavior of HSCs is inhibited by ROCK inhibitor treatment in a liver scenario, thus, resulting in the superior regeneration of liver [117]. Furthermore, the Src–JNK–YAP/TAZ pathway plays an important role in the mechanosensing of osteoblastic cells [118]. Other signaling pathways related to scaffold biocompatibility needs to be explored.

### 4.3. Migration

A key biological property of living cells is cell migration, which plays a critical role in establishing and maintaining the proper organization of multicellular tissues [119]. Cell migration is associated with the continuous assembly and disassembly of focal adhesions (FAs) which mediate the signal transduction or adhesion to the ECM, and which are regulated by specific signaling pathways [120,121]. In tissue engineering, cell migration in scaffolds is important for the regeneration of tissue defects [122]. Stem cells often have a homing ability which enables them to migrate into the injured sites for tissue regeneration. The migratory trajectories of cells manifest directed movement by “topotaxis” [123]. Cell migration is a complex process that can be influenced by a variety of factors, which can roughly be divided into the following two categories: the chemical factors and the mechanical factors [124]. We mainly focus on the mechanical effects of scaffold topographic orientation on cell migration. 

Patel et al. developed scaffolds with different topographic orientations loaded with cytokines and matrix protein onto the fibers for the regeneration of skin tissue. They found that aligned scaffolds significantly induced the outgrowth of neurons and enhanced the migration of skin cells as compared to random scaffolds [125]. Tan et al. used PLGA solution to fabricate scaffolds with electro-hydrodynamic jet (E-jet) 3D printing technology to culture fibroblasts. Results showed that cells migrated faster when the scaffold topographic orientation was aligned with the cell angles [126]. To mirror the irregular shape of the wound, Xie et al. designed a novel scaffold with radially aligned nanofibers by electrospinning for the regeneration of dural tissue [101]. For dural fibroblasts, aligned scaffolds induced faster cellular migration to the center of scaffolds as compared to random scaffolds, which resulted in a high population of cells throughout the aligned scaffolds [101]. To further promote cell migration by tuning physical factors, Yang et al. produced scaffolds with graded channels (e.g., gradual changes in porosity, stiffness, and topology) by freeze-casting hydroxyapatite slurries [122]. They compared the migration behavior of rat mesenchymal stem cells on freeze-dried scaffolds with the following three different topographic channels: random, aligned and graded. It was observed that graded scaffolds significantly enhance cell migration as compared to random and aligned, whereas the aligned scaffolds resulted in enhanced migration as compared to the random scaffolds [122]. This may be caused by the “capillary effect” of the graded channels that accelerate the exchange of fluid and metabolic products of cells [127]. The “nutrients gradient” caused by graded channels may be another important explanation [128]. Overall, compared to random scaffolds, the features of aligned topography promote cell migration, thereby increasing the number of cells in scaffolds and repair sites. In addition, scaffolds with graded channels further increase cell migration as compared to aligned orientation. 

The topographic orientation of scaffolds regulate cell migration through a variety of cellular pathways. For example, in migration of cells in bone and teeth tissue, it is proven that the Wnt/β-catenin signaling pathway is important [129]. In nervous tissue, the Piezo-1 (also named FAM38A), which is a mechanosensitive ion channel, and a series of membrane curvature sensing proteins, such as the Bin/amphiphysin/Rvs (BAR) domains, are also shown to be important [130,131].

## 5. Application of Scaffold Topographic Orientation for Tissue Regeneration

### 5.1. Vascular Tissue Regeneration

Blood vessels have a lumen through which blood flows. Its wall can be divided into the following three layers: tunica intima, tunica media, and tunica adventitia. The inner layer, tunica intima, is covered with endothelial cells (ECs), while the middle layer is intricately arranged with lots of smooth muscle and elastic fibers, and the outermost layer, tunica adventitia, is built up of collagen and elastic fibers [82]. All these structures are specifically arranged in order to better resist stress and load from all directions [82]. Vascular-like atherosclerotic diseases are common in the elderly population [82]. Nowadays, the typical treatment for these diseases is vascular bypass surgery, which is not suitable for small diameter vessels because it tends to trigger thrombus and intimal hyperplasia [132]. Currently, tissue engineering is a new approach for vascular regeneration. The topographic orientation of scaffolds in vascular regeneration affects the repair of tunica intima [133]. 

**Figure 3:**
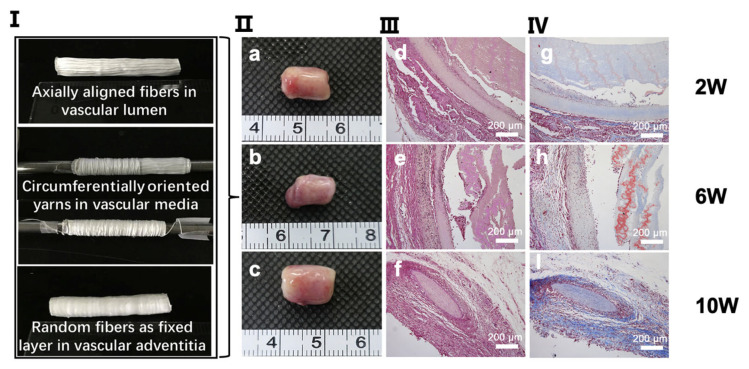
(**I**). The three-layer vascular scaffold consists of axially aligned PLCL/COL fibers in the inner layer, circumferentially oriented PLGA/SF yarns in the middle layer, and random PLCL/COL fibers in the outer layer as the fixed layer. The scaffolds were subcutaneously embedded in mice for 2W, 6W, and 10W. (**II**). The general morphology of the transplanted scaffolds after subcutaneous embedding in mice for 2 weeks (**a**), 6 weeks (**b**) and 10 weeks (**c**). (**III**). H&E staining images of the transplanted scaffolds after subcutaneous embedding in mice for 2 weeks (**d**), 6 weeks (**e**) and 10 weeks (**f**). (**IV**). Masson’s trichrome staining images of the transplanted grafts after subcutaneous embedding in mice for 2 weeks (**g**), 6 weeks (**h**) and 10 weeks (**i**). In vitro studies showed that aligned PLCL/COL fibers and PLGA/SF yarns promoted endothelial and smooth muscle cell proliferation and alignment along the fiber direction, respectively. (Reproduced from [142], Copyright © 2018 Elsevier Ltd.).

The ECs are classified as simple squamous epithelial cells that cover not only the inner surface of blood vessels but also lymphatic vessels to maintain vascularity, blood fluidity, etc. [134,135]. In the field of tissue engineering for vascular regeneration, the formation of a confluent EC monolayer in the lumen is crucial when implanting small diameter grafts [136,137]. The failure of implantation is often caused by thrombosis and hyperplasia due to insufficient endothelial cell coverage [138,139]. In the process of endothelial formation, the topographic orientation of scaffolds plays an important role. Li et al. found that ECs seeded on aligned scaffolds had a longer axis and a longer cell skeleton as compared to random scaffolds [102]. In addition, a larger nuclear aspect ratio was observed in aligned scaffolds, which can enhance cell activations, such as proliferation, viability, and adhesion, to better form the confluent endothelial layer by regulating specific gene expression [102]. The mechanism of why ECs cultured on aligned scaffolds show better adhesion and expansion ability as compared to ECs on random scaffolds is speculated to be because the aligned topographic orientation mimics the native tissue of aligned collagen fibers [140,141]. It has been discovered that ECs in aligned scaffolds are more regularly aligned in one direction [102,142] (Figure 3) and, furthermore, are shown to be aligned along the direction of blood flow in vivo [133]. Chernonosova et al. compared the influence of different topographic orientations of PCL scaffolds on HUVEC ECs for blood vessel regeneration [143]. They found that cells on aligned scaffolds exhibited elongated shapes with a substantial expression of VE-cadherin around the cells, while no effects on either cell morphology or overexpression was found in random scaffolds from in vitro experiments. In comparison to static conditions of cell cultivation, results from dynamic conditions have reported that more cells adhered to the surface of aligned fibers as compared to random fibers at a shear of 20 dyn/cm^2^ and 40 dyn/cm^2^ [143]. To summarize, ECs seeded on aligned scaffolds appear more like ECs in the human body, with a higher ability to resist blood flows, attach to matrices, and form a continuous endothelial layer. 

### 5.2. Skin Tissue Regeneration

Skin, the largest organ of the human body, accounts for 7% of the body weight and has an important protective effect on the organism [144]. The skin is composed of two parts, namely the epidermis and dermis. The epidermis can be divided into five layers, namely the stratum corneum, the hyaline layer, the granular layer, the spinous layer, and the basal layer. All five layers are composed of keratinocytes at different stages. There are no blood vessels within the epidermis but there is an abundance of free nerve endings [84]. Dermis provides structure and elasticity to the skin and is composed of connective tissue including intricate fiber networks and mucopolysaccharides [145]. There are many causes of skin tissue injury in life, including burns, contusions, hematomas, or diseases. Common diseases, such as diabetes have greatly increased the incidence of chronic wounds, making skin tissue injuries of high social significance [146,147]. However, the skin has a limited ability to regenerate and, thus, tissue engineering for skin regeneration has received extensive attention.

To better resist tension in all directions, the fibers in the dense connective tissue of the dermis are disorganized. Thus, in order to mimic the natural ECM of skin tissue, the most logical approach was to use random scaffolds [148]. Random scaffolds have been verified by many studies to have a highly desirable skin repair effect. Jha et al. treated the porcine skin injury model with collagen electrospinning scaffolds crosslinked with glutaraldehyde, while Said et al. treated the rat skin injury model with fusidic acid (FA)-loaded PLGA electrospinning scaffolds. These studies by Jha et al. and Said et al. concluded that, based on in vitro and in vivo tests, random scaffolds induced a more efficient wound healing process with less inflammatory response [149,150]. Furthermore, Coskun et al. compared the randomly oriented electrospun poly (vinyl alcohol)/sodium alginate for wound dressing with commercially available drugs to treat skin injuries (such as tulle grass, Smith & Nephew, Lohmann, etc.) and concluded that a random scaffold resulted in better epithelization, epidermis characteristics, vascularization, and formation of hair follicles as compared to the commercially available drugs [151]. All data support the idea that random scaffolds have an excellent effect on the repair of skin. Although the fibers in the dermis of the skin are disorganized, many studies have reported that the arrangement of these fibers is related to Langer’s lines, which are topological lines drawn on a map of the human body [152,153]. In fact, some experiments using aligned scaffolds for skin repair have been studied. Although the effect of promoting wound repair was not as significant as the random scaffold, the aligned scaffold was able to reduce neurite outgrowth and enhance the skin cell migration [125,154]. Overall, in tissue engineering for skin wound healing, random scaffolds are more stable and comprehensive. Nevertheless, for parts of the skin exposed to stress in a single direction or in the case of higher requirements for nerve fiber repair, it might be that scaffolds with aligned topographic orientation have advantages.

### 5.3. Neural Tissue Regeneration

The nervous system is widely distributed in the body, consisting of the central nervous system (CNS) and peripheral nervous system (PNS). The CNS consists of the spinal cord and the brain. The PNS is comprised of sensory neurons that are responsible for transmitting peripheral stimuli to CNS and motor neurons that excite skeletal muscle movement [155]. Nerve tissue is composed of nerve cells (neurons) and glial cells. Neurons, consisting of a cell body (soma), axons, and dendrites, can receive stimuli, integrate information, and transmit impulses. Glial cells, of which there are 10–50 times as many compared to neurons, play an important role in support, protection, nutrition, and insulation [156]. In addition to traumatic nerve damage, some senile neurodegenerative diseases have become more and more common, such as Alzheimer’s disease, Parkinson’s disease, etc. The development of tissue engineering scaffolds provides a strategy for the repair of these nerve tissues. However, the conventional theories suggest that neurons cannot regenerate by mitosis, so the regeneration of nerve tissue is a difficult challenge [157]. Existing tissue engineering methods use polymeric scaffolds to simulate or replace the structure and function of ECM, since these have powerful regenerative and cellular support capabilities that can develop an alternative nerve tissue regeneration pathway [157]. To summarize, the purpose of tissue engineering for neural tissue is to replace traditional strategies, such as autologous, allograft, and xenograft, as these strategies are limited in their use due to lack of donors [155,158]. By reviewing the current literature, we found that scaffolds with aligned topographical orientation are more suitable for the regeneration of neural tissue.

When used for axon regeneration, nerve guiding channels are usually made into hollow tubes or porous foam rods [155]. On the inner surface of these tunnel scaffolds, the intraluminal channels, the oriented nerve substratum, and biodegradable porous can be designed and growth factors or supporting cells can be loaded, which make the scaffold an effective nerve conduit [155]. Schwann cell is a type of glial cell which is the main component of the myelin sheath on the axons of myelinated neurons in the peripheral nervous system [159]. Schwann cells are very crucial for the repair of nerve tissue. For myelinated neurons, the regeneration of the broken axon often depends on the state of the surrounding Schwann cells [159]. In terms of tissue engineering, Yang et al. observed that Schwann cells seeded in random scaffolds proliferated faster than the aligned scaffold group, which might be explained by the high porosity and rough surface of the random scaffolds [103,160]. Nevertheless, it has been reported that Schwann cells growing on aligned scaffolds with “contact guidance” of the aligned topography both provided better guidance cues for the growth of neurons and extension of axons, which contributes to nerve regeneration [103]. Previous studies have confirmed that aligned fibers could guide the direction of regenerating axons and migrating Schwann cells. Wang et al. found that the majority of neurite outgrowth and Schwann cell migration were unidirectionally parallel to the aligned fibers on the aligned fibrous membranes, while neurite outgrowth and Schwann cell migration were random on random fibrous membranes [115]. The mechanism of why an aligned topographic orientation better stimulates peripheral nerve regeneration as compared to random topographic orientation might be explained by their differential modulation impact on macrophage phenotypes in ECM. It is reported that an aligned orientation induced macrophages to the proinflammatory phenotype (M2 type) that subsequently could enhance the migration and proliferation of Schwann cells in vitro, whereas random orientation induced macrophages to the proinflammatory phenotype (M1 type) [161]. In terms of nerve function, nerves repaired by aligned scaffolds have great compound action potentials (CAP) measurements, while there was no CAP detected in nerves repaired by random scaffolds. Use of an aligned scaffold, in new neuromuscular junctions, resulted in significantly better function as compared to the random scaffold [162]. Regarding materials, it has been demonstrated that both the aligned and random scaffolds composed of PCL/gelatin were more beneficial for the bioactivity and morphology of Schwann cells than those made up of PCL [103]. In conclusion, although nerve conduits with random topographic orientation may promote proliferation of Schwann cells, nerve conduits with aligned topographic orientation are more feasible for the overall repair effect of the function of nervous system.

Tissue engineering scaffolds for neural tissue repair using cells other than Schwann cells have also been reported. Rat neuronal-like cell lines show a better cellular phenotype, higher cell proliferation, and improved neurite outgrowth on aligned scaffolds as compared to random scaffolds [97]. The use of neural stem cells (NSCs), which can differentiate into intermediate progenitor cells and subsequently neuroblasts, has become popular for regeneration of the nerve system. They inhabit certain niches, such as the subventricular zone (SVZ) and dentate gyrus subgranular zone (SGZ) of the hippocampus [163]. It has been reported that more NSCs in aligned scaffolds expressed the immature neuronal marker Tuj1 with the presence of RA/FBS as an inducer, indicating that aligned topography favors the yield of neuronal progenitors [107]. The reason for this might be that the substrate topographic orientation favors neuronal cell morphology and, thus, promotes cell survival by activating the canonical β-catenin/Wnt signaling pathway which is pivotal in neurogenesis [107]. 

### 5.4. Bone Tissue Regeneration

Bone, which originates from the mesenchyme during the embryonic period, is the mechanical support of the human body. The main body of bone is bone tissue, which consists of bone matrix (collagen fibers, amorphous matrix as organic matter, and hydroxyapatite crystals as inorganic matter) and bone tissue cells. Bone tissue is strong and hard and, therefore, plays an important role in supporting and protecting the body during movement [164]. Bone injury has always been a problem for human health. The most common types of clinical injuries are bone defects caused by trauma, tumors, infection, or bone diseases [165]. Bone remodeling involves the removal of old or damaged bone by osteoclasts (bone resorption) and the subsequent replacement of new bone formed by osteoblasts (bone formation). Normal bone remodeling requires a tight coupling of bone resorption to bone formation in order to guarantee no alteration in bone mass or quality after each remodeling cycle [166]. However, due to lack of vascularity and nerves, the regeneration process of bone defects is very slow and difficult. Based on the fact that bone heals very slowly, bone tissue engineering is attracting more and more attention. It has been reported that bone tissue engineering is better than the approaches used traditionally [167]. Furthermore, topographical cues provided by micropatterns on material surfaces have been demonstrated to control multiple cellular behaviors. For example, the potential benefits of incorporating osteon-like concentric microgroove patterns on the surface of scaffolds for bone repair have been highlighted [168]. Satisfying repair of bone tissue is closely related to osteocytes and the bone matrix. Cells in bones include osteoprogenitor cells, osteoblasts, osteoclasts, and osteocytes [169]. 

Osteoprogenitor cells are the common stem cells of bone tissue and are located in the inner lining of the periosteum. Their differentiation fate depends on the location and nature of the injury. During bone growth, remodeling, or fracture repair, osteoprogenitor cells are active, and proliferate and differentiate into osteoblasts [164]. Badami et al. found that aligned fiber scaffolds with diameters of 0.5–2 microns could induce the parallel arrangement of osteoblasts and differentiation of osteoprogenitor cells into osteoblasts [170]. When cells were planted and grown on the aligned fiber scaffold, it was seen that cells usually adhered to the fiber in clusters along the fiber direction. A superimposed phase contrast imaging of the fiber substrate revealed that focal adhesion contacts frequently occurred as clusters along the fibers [171]. In a study of microtubular architecture of scaffolds, Ma et al. found that osteocytes were arranged along the morphological direction of the fiber, thus, enhancing the physiological activity of the osteocytes [172]. On the aligned scaffold, the morphological evolution was more obvious and could effectively promote the differentiation of osteoprogenitor cells into osteoblasts [171,172]. In conclusion, osteoprogenitor cells, as common stem cells of cartilage and bone tissue, play an important role in the repair of bone injury. The limited knowledge, based on current studies, suggests that scaffolds with the architecture of aligned microtubules can improve the growth, arrangement, and differentiation of bone progenitor cells. 

Osteoblasts, differentiated from osteoprogenitor cells, are distributed on the surface of bone tissue and arranged in a single layer, low columnar or irregular form. They play an important role in the treatment of bone injury due to their active differentiation and secretory capacity [164]. As an effective way to promote bone regeneration, scaffold implantation can mimic the layered structure of extracellular bone matrix in vivo, thereby inducing osteoblast proliferation and differentiation [164]. Many studies have shown that the distribution of osteoblasts differs depending on the topographic orientation of scaffolds. Lee et al. reported that both the alignment of osteoblasts on the scaffolds and the apatite c-axis orientation were enhanced in scaffold with more aligned fibers, which indicates better bone repair [173]. It has also been demonstrated that the arrangement of osteoblasts and the resulting ECM was influenced by the external environment [174]. Cheng et al. found that aligned and randomly arranged fiber scaffolds, obtained by electric clip (PLLA/PCL) and electric spray, had different mechanical properties and different effects on the growth activity of osteoblasts. Aligned fiber scaffolds showed improved mechanical properties, better cell coverage, and higher bioactivity as compared to random fiber scaffolds [175]. Thus, osteoblasts, as important cells in the process of bone regeneration, can be controlled by adjusting the topographical orientation of scaffolds. Osteoblasts seeded on aligned scaffolds have higher gene expression and biological activity as compared to cells seeded on random scaffolds. It has been reported that global DNA methylation of 5-methylcytosine, 5-hydroxymethylcytosine, and N-6 methylated deoxyadenosine of human osteoblasts on aligned scaffolds is highly activated as compared to on random scaffolds. Thus, epigenetics may be related to the effect of the topographic orientation on osteoblasts [176].

Osteoclasts are a kind of wandering multinucleated cells with a strong osteolytic capacity. Osteoclasts and osteoblasts complement each other and participate in bone growth and remodeling [164]. Interestingly, Detsch et al. found that osteoclasts rule osteoblasts in different ways, depending on their stage of differentiation [177]. Thus, co-cultures of osteoclasts and osteoblasts could be beneficial to the microenvironment of the bone scaffold, given that osteoclasts are involved in functional bone regeneration. Scaffolds should be completely degraded within an adequate period. The degradation of synthetic bone substitute materials involves both chemical dissolution (physicochemical degradation) and resorption (cellular degradation by osteoclasts). Osteoclasts also play a crucial role in bone remodeling, which is essential for the regeneration of bone defects [177]. Osteoclasts can differentiate, degrade fibrinogen (Fg)-3D, and produce factors, such as TGF-β1, that promote MSC osteogenic differentiation [178]. Thus, osteoclasts co-cultured with osteoblasts in the appropriate scaffolds could show a marvelous result in bone defect regeneration. 

The bone matrix in bone tissue is the calcified ECM. It includes organic and inorganic components with a low density of water [164]. The organic composition consists of a large number of collagen fibers (mainly composed of type Ⅰ collagen) and a small amount of amorphous matrix (mainly composed of proteoglycans and their complexes) [164]. The inorganic components, also known as bone salts, are mainly composed of hydroxyapatite crystals, derived from matrix vesicles secreted by osteoblasts [164]. The mechanical properties of bone tissue are strongly correlated with the degree of BAp c-axis orientation, which is one of the indices of bone quality. Notably, the bone quality is more related to the mechanical properties of bone tissue than the bone quantity [173]. Since fiber orientation greatly influences cell growth and related functions, the orientation and type of collagen fibers have a major impact on the phenotypic expression of the osteoblasts and the properties of the matrix. Fee et al. found that fibroblasts on the aligned nanofiber scaffolds were aligned parallel to the fibers, and their gene expression was upregulated through actin production, actin polymerization, and focal adhesion formation [179]. Additionally, scaffolds with aligned nanofiber were also found to be able to upregulate the expression of osteogenic markers, such as runt-related transcription factor (Runx-2), type I collagen, alkaline phosphatase (ALP), bone sialoprotein (BSP), and osteocalcin (OCN) [180]. Overall, the production of the matrix is closely related to the function of osteoblasts. Osteoblasts on aligned scaffolds are elongated and parallel to the direction of fiber alignment, which results in upregulation of cytoskeleton-related gene expression, while collagen matrix production oriented in the direction of cellular alignment, and the c-axis of the deposited apatite crystals, indicate a preferential alignment along the direction of the collagen matrix [181,182,183]. 

In summary, compared with random scaffolds, aligned scaffolds can induce a more rational arrangement of osteoprogenitor cells and osteoblasts with enhancement of their biological activity, resulting in more of a matrix and a tighter bone structure. Furthermore, the influence of aligned scaffolds on osteoclasts also contributes to the remodeling of bone tissue. In conclusion, aligned scaffolds are more suitable for bone tissue regeneration as compared to random scaffolds.

### 5.5. Articular Cartilage Regeneration

Articular cartilage is a special kind of hyaline cartilage that is critical to the normal function of synovial joints. It consists of cells (including chondroblasts and chondrocytes) and amorphous ECM. Chondrocytes are the main cells in cartilage and are distributed as single cells or as a homogenous group in the ECM, which is composed of interstitial fluid, collagen fibers (mainly type II collagen fibers) and proteoglycans [184]. Chondrocytes are responsible for the synthesis and maintenance of these ECM components. The perichondrium covers the surface of the articular cartilage [184]. Cartilage is an avascular and non-nervous tissue without lymphatic vessels, relying on the diffuse movement of surrounding cells to provide nutrients. Due to the slow diffusion rate and a small number of cells, the self-repair of cartilage is slow and self-limited [185,186,187]. At present, osteochondral injury is one of the most common skeletal system diseases in the world, and is related to age, trauma, genetic factors, etc. The incidence of cartilage damage is also expected to grow with an increasingly older population. Thus, an approach for successful cartilage repair would be of great significance both sociologically and economically [188,189]. However, the results obtained by the current methods for clinical repair (such as microfractures, drilling, etc.) are not very satisfactory. Most of the newly formed tissues obtained by the current methods are inferior to the native with mechanical fibrocartilage rather than hyaline cartilage [190,191]. Thus, tissue engineering to repair cartilage damage and regenerate high-quality hyaline cartilage containing a large number of type II collagen fibers is warranted and has a great application potential [192]. 

In experiments by Accardi et al. electrospinning was applied to obtain PCL scaffolds with different fiber orientations [193]. In a test of mechanical properties, they found that the aligned fibrous scaffolds had the highest tensile modulus and the frictional response of cartilage tissue was not affected by the orientation of the scaffolds. This implies that the mechanical properties of aligned scaffolds were superior to those of random scaffolds and could be used in the early protection of cells from critical compression [193]. However, since collagen damage might be caused by excessive shear and excessive strain along collagen fibers [194], the fiber orientation of aligned scaffolds should be in the direction of the shear or motion. Based on the isotropy of random scaffolds, it may possess a better topographical surface for friction and wear in applications. Although the two scaffolds have their advantages and disadvantages, Mario et al. believe that the aligned scaffolds have better application prospects [193]. 

Jia et al. used freeze-drying to prepare scaffolds, and the constructs were placed on the back of nude mice after inoculation with BMSCs. Observing the characterization of scaffolds after the experiment, they found that the pores in the aligned scaffolds exhibited a parallel-arranged microtubule structure, similar to the structure of natural cartilage tissue that guided BMSCs to adhere and align in the vertical direction [195]. The pores in the random scaffolds were randomly and uniformly distributed [195]. Because the microtubule structure facilitates nutrient exchange and transport of metabolic wastes in the early stage, the cells in aligned scaffolds proliferated more vigorously than those in the random scaffolds at 3–9 days [195]. After excluding the interference of cell number and biochemical composition, they further concluded that the excellent mechanical properties of the constructs were related to their directional arrangement, which is similar to the orientation of the collagen bundles formed by chondrocyte secretion. However, the models applied are relatively limited and, thus, animal models of full-thickness cartilage defects are warranted to test the above conclusions [195]. 

**Figure 4:**
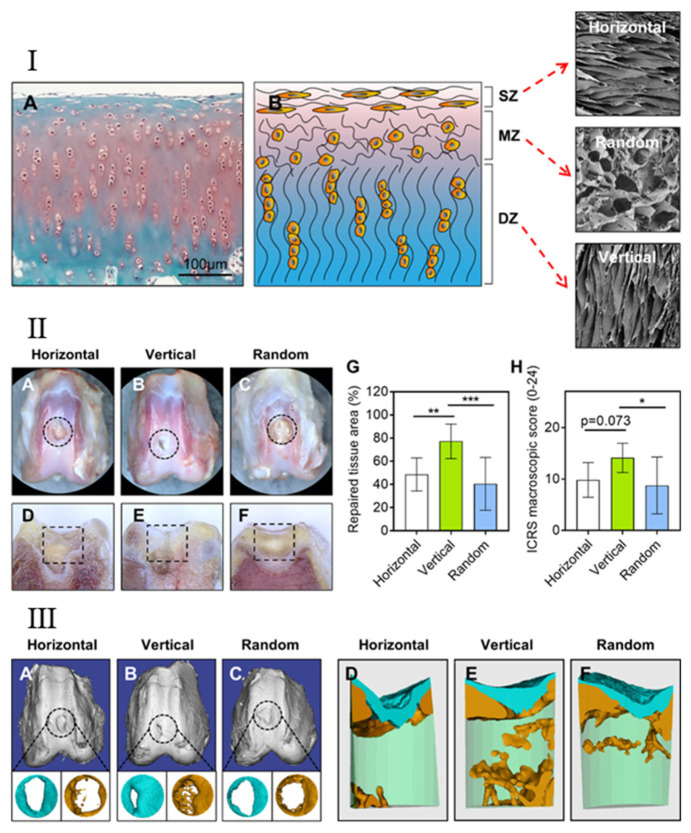
(**I**). Schematic illustration of the ECM structure of cartilage. The ECM of cartilage is divided into three layers, as follows: the horizontally aligned, randomly arranged, and vertically aligned from the superficial layer to the deep layer. Abbreviations are as follows: SZ, surface zone; MZ, middle zone; DZ, deep zone. (**II**). The horizontal, vertical, and random scaffolds fabricated using controlled directional freeze-drying were implanted into the rabbit osteochondral defect model, and the repair efficacy was evaluated after 12 weeks. There was a lot of cartilage neotissue on the surface of the vertically arranged scaffold as well as in the repaired tissue area, and the average ICRS macroscopic scores were higher than those of the other two groups (* *p* < 0.05, ** *p* < 0.01, *** *p* < 0.001). (**III**). Evaluation by micro-CT and 3D reconstruction. The vertically aligned scaffold group showed superior cartilage and subchondral bone repair (Reproduced from [196], American Chemical Society).

Recently, our group fabricated biomimetic scaffolds with three different cartilage ECM-like architectures, i.e., horizontal, random, and vertical arrangement by directional freeze-drying technology (Figure 4) [196]. We subsequently implanted the three different scaffolds into a rabbit osteochondral defect model and made evaluations after 12 weeks. We found that the vertically aligned scaffolds promoted cartilage and subchondral bone repair better than the other two scaffolds. This may be due to the better hydrophilicity of the aligned scaffolds, which can absorb more nutrients in the exchange with the surrounding fluid environment and recruit more BMSCs after blood infiltration of the bone marrow [196]. In addition, the aligned scaffold possessed a higher pore aspect ratio, which is expected to enhance capillary phenomena and fluid movement, thereby contributing to improved tissue regeneration. Meanwhile, compared with the horizontally arranged scaffolds, the vertically arranged scaffolds provided parallel holes along the bone axis, thus, promoting the upward migration of BMSCs and facilitating osteochondral regeneration [196].

In summary, although random scaffolds have advantages in some aspects, such as superior friction resistance [193], aligned scaffolds are the preferable choice for articular cartilage regeneration. Aligned scaffolds are more in line with biomimetic strategies and can promote chondrocyte migration and proliferation [195,196] and, thus, have more comprehensive and stable applications in cartilage regeneration.

### 5.6. Ligament and Tendon Regeneration

Ligaments and tendons are dense regular connective tissues with a large number of collagen fibers arranged in bundles aligned in the direction of stress [197]. Because ligaments and tendons bear a lot of stress to mediate muscle movement, these tissue are exposed to a high risk of injury. Both athletes and non-athletes are commonly afflicted by such injuries, which are tricky to repair successfully in the clinic due to restricted blood supply, which result in poor regeneration capability [198]. Since traditional treatments are insufficient, scaffolds for tissue engineering can be an attractive alternative for ligament and tendon tissue regeneration. For ligament and tendon tissue engineering, it is known that the topographic orientation of scaffolds influences the regeneration effect. 

**Figure 5:**
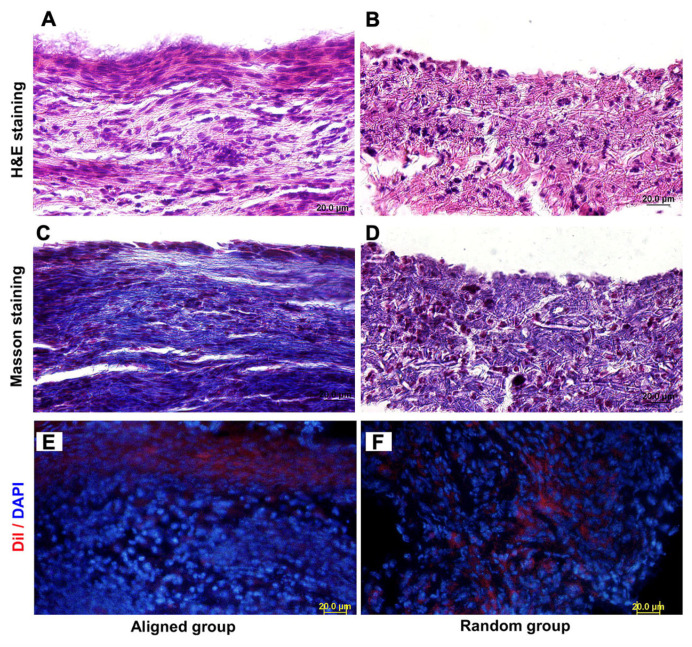
As compared to random scaffolds, aligned scaffolds can better promote tendon tissue regeneration in vivo. Aligned scaffolds induced a spindle-shaped morphology of cells (**A**) and dense connective tissue (**C**) in vivo, resulting in a highly organized structure similar to the native tendon matrix, in contrast to the random scaffold group that resulted in round cells (**B**) and a loose, disorganized matrix (**D**). Implanted hTSPCs participated in the regeneration of new tissue on both aligned (**E**) and random scaffolds (**F**) (Reproduced for [80], Elsevier Ltd.).

Tendon stem/progenitor cells (hTSPCs) are obtained from tendons. This is a group of cells with common characteristics with stem cells, such as self-renewal, clonality, and multi-directional differentiation [199]. Since hTSPCs have the potential to differentiate into chondrogenesis, adipogenesis, and osteogenesis, they are an ideal group of cells to use for the regeneration of the tendon. As shown in the Figure 5, when contrasting hTSPCs seeded on aligned and random electrospinning scaffolds, Yin et al, found that both the distribution of cells and the orientation of collagen fibers were more oriented along the nanofibers of scaffolds in the aligned group as compared to random scaffolds [80]. In addition, cells grown on aligned scaffolds exhibited a morphology similar to the typical phenotype of fibroblasts, i.e., spindle-shaped and elongated [80]. The cells observed on the random scaffolds appeared with a stellate-pattern. The difference in morphology between random and aligned scaffolds might be explained by the influence of nanofibers on cell–matrix interactions [80]. Since cells with more physiological morphology often have increased cell viability, it is reasonable to assume that the repair effect of aligned scaffolds is better as compared to random scaffolds. In addition, based on specific gene expressions it has been discovered that the topographic orientation of scaffolds could significantly influence the differentiation of hTSPCs. When hTSPCs were seeded on the aligned scaffold, they were induced to teno-lineage even in an osteogenic induction medium, while cells on the random scaffold were induced to osteo-lineage [80]. Similar results have also been confirmed in vivo [200]. The mechanism of why an aligned scaffold is better for tendon repair as compared to random scaffold may be that cells growing on the aligned scaffold have significantly increased integrin subunits (α1, α5, and β1) which further control gene regulation, the cytoskeleton, and various cellular behaviors via downstream signaling cascades [80]. Pilipchuk et al. developed aligned and random poly(ε-caprolactone) (PCL) scaffolds specific to human ligament progenitor cells by 3D printing. They implanted scaffolds loaded with human ligament cells and fibroblasts into a murine model [201]. Results showed that the aligned scaffolds better increase tissue alignment as compared to the random scaffolds. Aligned scaffolds with 30 μm deep grooves significantly enhanced the thickness of collagen fibers, overall alignment of cells, and the elongation of nuclei as compared with aligned scaffolds with 10 μm deep grooves [201]. These results confirm the better ability to form aligned bone–ligament–cementum complexes in vivo with aligned scaffolds as compared to random scaffolds made by 3D printing [201]. To sum up, aligned scaffolds are more suitable for the regeneration of wounded ligament and tendon tissue.

### 5.7. Cardiac Tissue Regeneration

The heart, which is the central organ in the human circulatory system, pumps blood throughout the whole body to maintain circulation. The wall of the heart is composed of the endocardium, myocardium, and epicardium from the inside to the outside. The myocardium is composed of two major tissues containing specific cell types, as follows: the myocardial tissue comprised of cardiomyocytes (CMs) and the ECM comprised of cardiac fibroblasts (CFs) [202]. Cardiovascular disease (CVD) is the leading cause of death in the world [203]. In recent years, due to the increased aging of the population and the stress of life, myocardial infarction and myocardial fibrosis caused by atherosclerosis have become more common. Among the various methods to treat myocardial defects, tissue engineering is an emerging therapeutic approach which has attracted widespread attention. Previous studies have shown that most cell types responded to the topographical features of the underlying substrate, especially the topographic orientation of the scaffold [94]. Thus, this review will compare the effect of aligned and random scaffolds on CMs and CFs for cardiac regeneration.

The CMs have periodic horizontal stripes and darker staining, called intercalated discs, at their junctions. The intercalated discs have many gap junctions, which make the entire myocardium function to appear as a syncytia [204]. When tissue engineering technology is used to repair myocardial cell defects, most studies have reported that aligned scaffolds were more suitable than random scaffolds. The CMs on aligned scaffolds were found to grow along the nanofibers of the scaffold, and their nuclei appeared elongated, while CMs on random scaffolds were disoriented [94,202]. This discovery may suggest that “contact guidance” of cells in aligned topography can influence the regenerating tissues to reproduce cardiac anisotropy in vivo. Using fluorescent staining, Orlova et al. and Kharaziha et al. found that the α-actin of MCs grown on randomly oriented nanofibers exhibited partial arrangement without uniform anisotropy, while MCs grown on aligned nanofibers were arranged evenly. These results indicate that the cellular interactions were the significant factor to affect cell alignment. They also discovered that MCs grown on aligned scaffolds had stronger contractility [202,205]. It has been reported that the proliferation of MCs is higher on the aligned scaffolds as compared to random scaffolds [206]. Meanwhile, previous studies have shown that aligned scaffolds promote the differentiation of stem cells towards CMs and the maturity of CMs as compared to random scaffolds [202,206,207]. In addition, regenerative cardiac tissues grown on aligned scaffolds had better electrical conductivity, namely more efficient driving, higher beating rates, and greater synchronization [202,203]. Although the aligned scaffolds have outstanding anisotropy, they still have some drawbacks, mainly that they have weak intrinsic mechanical properties. To solve this problem, Eom et al. developed a random/aligned hybrid nanofiber scaffold for cardiac regeneration, which resulted in satisfying test results [208].

The ECM of myocardial tissue exhibits a well-defined three-dimensional (3D) fibrous structure that plays an important role in maintaining the normal function of the heart [209]. The CFs, the main cell phenotype in ECM, can secrete fibers and maintain the stability of the microenvironment in CMs. In natural cardiac tissue, CFs affect the function of CMs through autocrine and paracrine signaling [210]. Therefore, maintaining the number and function of CFs has an important impact on the repair effect of tissue engineering. Overall, aligned nanofiber scaffolds are more suitable for the regeneration of CFs. It has been demonstrated that CFs in random scaffolds exhibited random orientation and larger cell size, while CFs on aligned scaffolds showed a more spindle-shaped morphology along the direction of nanofibers that could mirror a better cellular state of CFs. In addition, CFs exhibited a better viability and proliferative ability on aligned scaffolds [202]. 

In summary, scaffolds with aligned topographic orientation are more suitable for cardiac tissue regeneration as compared to random scaffolds. For MCs, aligned scaffolds better promote their biological behaviors, such as proliferation, differentiation, contractility, and electrical conductivity. In addition, cardiac tissue repaired with an aligned scaffold is anisotropic, which is more in accordance with the principles of biomimetic. For MCs, aligned scaffolds are also more suitable than random scaffolds because MCs grown on aligned scaffolds possess higher biological activity and exhibit a more physiological cell-shape. Based on current knowledge, aligned scaffolds are more beneficial for cardiac tissue regeneration.

### 5.8. Cornea Regeneration

The cornea is an avascular tissue of the eye which plays an important role in providing optimal vision and protecting more delicate structures in the eyeball [211,212]. In recent years, traditional corneal transplantation has been the main treatment method to cure damaged cornea. However, even in many developed countries, it is difficult to get access to this surgery due to a lack of donors. Therefore, tissue engineering is becoming more and more popular for the treatment of corneal defects. The cornea is divided into five layers; from the outside to the inside are the epithelial layer of the cornea, the anterior elastic layer, the corneal stromal layer, the posterior elastic layer, and the endothelial layer [213]. There are two kinds of cells in the cornea, namely corneal epithelial cells and keratocytes [214]. 

The epithelial layer is the first important line of defense for the cornea against foreign invasion, and it is a non-keratinized squamous epithelium. The corneal epithelial cell layer grows quickly and is firmly bonded. It has great resistance to most bacteria and toxins. It can regenerate after damage and can be repaired within 24 h without leaving scars [215]. Thus, the presence of the corneal epithelium is very necessary for the normal physiological function of the cornea. When using tissue engineering to promote the regeneration of corneal epithelial cells, Yan et al. observed a better proliferation of corneal epithelial cells on random scaffolds as compared to aligned scaffolds [214]. In addition, they found that random scaffolds resulted in higher adhesion and viability, as well as a higher expression of keratin in cells [214]. Furthermore, it has been reported that the fiber arrangement of scaffolds could guide the extension of the cytoskeleton in cells [214]. On random scaffolds the corneal epithelial cells were randomly distributed with a large and flattened morphology, while cells on aligned scaffolds remained round without lamellipodia and failed to arrange in a specific way. The corneal epithelial cells also had higher cell density and biomarker protein expression in random scaffolds as compared to aligned, which indicates a better differentiation potential [212,214,216]. Thus, randomly oriented scaffolds are more suitable for the regeneration of corneal epithelial cells.

**Figure 6:**
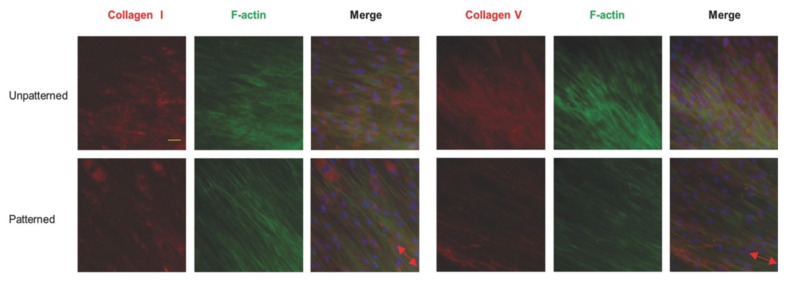
Immunofluorescence staining showed that collagens I and V, the main ECM of the corneal stroma, were well aligned on the patterned silk surface, thus, mimicking the ECM of the native cornea. The red arrows indicate the groove direction of the patterned films. Scale bar = 100 µm. (Reproduced from [218], Wiley-VCH GmbH, Weinheim, Germany).

Corneal keratocytes, also known as corneal fibroblasts, are specialized fibroblasts residing in the stroma of the cornea. The avascular stroma is the main component of the cornea, comprising its middle, thickest layer that accounts for 85–90% of corneal thickness. It is composed mainly of collagen I and V fibers arranged into bundles called lamellae, in between which the sparsely scattered collagen-producing keratocytes are found. Keratocytes play the major role in maintaining transparency, regulating the healing of wounds, and synthesizing their components, especially after injuries and inflammation [217]. Any glitch in the precisely orchestrated process of healing may result in lost transparency and blindness. It is speculated that excessive keratocyte apoptosis may be a part of the pathological process in degenerative corneal diseases [217]. Thus, considering the central role of keratocytes in the cornea, much focus is aimed at keratocytes in corneal tissue engineering. Yan et al. found that keratocytes on aligned scaffolds proliferated better as compared to keratocytes on random scaffolds, which was opposite to corneal epithelial cells. Nevertheless, random scaffolds performed better in terms of adhesion and viability and had a higher expression of vimentin [214]. It has been reported that both random and aligned scaffolds were suitable for the proliferation of keratocytes. However, it should be mentioned that cells cultured on random and aligned scaffolds had a distinct impact on the morphology of keratocytes. Keratocytes cultured on aligned scaffolds exhibited a spindle-shaped morphology and were arranged in parallel, while cells cultured on random scaffolds appeared randomly scattered [214]. Similar phenomenon, i.e., that keratocytes responded to substrates with their microstructure, was also observed by Wu et al., when they adapted PVA/Collagen composite nanofibrous electrospinning scaffolds for cornea regeneration [211]. They found that both cell density and biomarker protein expression were higher in keratocytes cultured on aligned scaffolds as compared to random, indicating better differentiation [214]. Wu et al. also found the ECM produced by cells on aligned scaffolds was very similar to the natural matrix, which is conducive to achieving better physiological functions [211,216]. We have previously developed a biomimetic 3D corneal model with random and aligned silk fibroin membrane to study the topographical effects on the keratocyte phenotype and ECM formation (Figure 6) [218]. We found elongated and aligned F-actin, keratocyte-like cell morphology, and collagen I and collagen V production by keratocytes cultured on aligned membranes, which mimic the native cornea [218]. According to these results, aligned scaffolds are the preference for cornea tissue repair.

Obviously, different structures of the cornea have different propensities to the different topographies of scaffolds. Aligned scaffolds are more beneficial for corneal stromal regeneration. In contrast, corneal epithelial cells grow well on randomly oriented scaffolds, which is the preferred scaffold for the reconstruction of the corneal epithelium in corneal tissue engineering. This phenomenon may be due to the fact that cells on aligned scaffolds tend to grow into a spindle-shape favoring corneal cells, whereas cells on random scaffolds tend to grow into a polygonal shape for corneal epithelial cells [214]. The scaffold will also be conducive for cell proliferation when the topographic orientation of the scaffold is consistent with the cell shape and cytoskeletal tension [214]. For corneal tissue engineering, multiple layers of scaffolds with different microstructures should be fabricated for optimal corneal repair.

### 5.9. Skeletal Muscle Regeneration

Skeletal muscles are attached to the skeleton by tendons and are the most widely distributed muscles in the body [26]. Skeletal muscle is composed of a large number of fiber-like muscle cells with contractile capacity. Dense connective tissue covers the entire muscle to form the epimysium, which extends into the muscle to form the perineurium and divides the muscle into multiple muscle bundles. The connective tissue outside each muscle fiber is called the endomysium. Skeletal muscle is a voluntary muscle that can produce axial contractions, thereby driving joint movements [219]. For minor injuries, the skeletal muscle can regenerate. However, when faced with severe injuries and diseases, skeletal muscles cannot heal by themselves, resulting in a loss of function [220]. Tissue engineering is a promising method to repair muscles [220]. In order to better reach the goal of repairing skeletal muscle to its natural state, much focus has been directed towards the topographic orientations of scaffolds.

Uehara et al. fabricated scaffolds composed of electrospun nanofibers containing graphene oxide for skeletal muscle regeneration [221]. They found that skeletal muscle cells (C2C12) on random scaffolds followed a trend of random direction while cells on aligned scaffolds showed a specific direction consistent with the aligned nanofibers. Skeletal muscle cells had a great attachment to both aligned and random scaffolds [221]. A similar phenomenon was also found on electrospun polymer fiber scaffolds, i.e., that C2C12 murine myoblasts cultured on aligned scaffolds exhibited more elongated morphology as compared to random scaffolds. Cells cultured on aligned scaffolds also maintained a satisfying attachment, proliferation, and differentiation [222]. Additionally, Ahadian et al. discovered that C2C12 growing on hybrid GelMA-vertically aligned CNT hydrogels generated more functional myofibers when repairing skeletal muscle defects than GelMA hydrogels with randomly or horizontally aligned CNTs [223]. Lin et al. fabricated aligned and random scaffolds by freeze-drying and implanted them into rat leg muscle tissue to compare their in vivo muscle repair effects [105]. They found that muscle tissue in aligned scaffolds had a higher rate of regeneration and a more orderly regeneration direction with more expression of muscle actin and desmin as compared to muscle tissue in random scaffolds. This may be due to the abilities of aligned scaffolds to better resist the deformation and induce oriented internal fluid for superior cell migration [105].

In summary, aligned scaffolds are good for the restoration of skeletal muscles when the topographic orientation is consistent with the direction of the muscle bundle contraction. However, random scaffolds are absolutely not inferior. Future in vivo work is warranted to compare the regenerative effect of scaffolds with random and aligned topographical orientation, by observing molecular weight modulations, surface morphology changes, pH changes, mass loss, etc. [222]. 

### 5.10. Smooth Muscle Regeneration

Smooth muscle cells (SMCs) are long and fusiform, widely distributed in the walls of the digestive tract, respiratory tract, and blood vessels. Tissue engineering technology often creates a tubular scaffold to repair smooth muscle damage [224]. Wang et al. developed a PLLA/PDMS tube loaded with SMCs for regeneration by using the electrospinning technique [225]. Through electron microscopy observations and gene expression analysis, they showed that SMC planted on the aligned fibers developed into a healthier phenotype with a fusiform shape, also regarded as a typical contractile phenotype, while the morphology of SMCs on the random fibers was epithelioid or rhomboid which is typical of the pathogenic synthetic phenotype [225]. Jia et al. developed aligned and random PU/Coll nanofibrous scaffolds for vascular regeneration [226]. They found that aligned scaffolds showed better anisotropic wetting performance, mechanical properties, morphological orientation, and protein promotion of SMCs as compared to random scaffolds [226]. Kobayashi et al. used orthogonally oriented scaffolds with aligned fibers to repair intestinal tracts and achieved a good repair effect [227]. In summary, for smooth muscle repair, aligned scaffolds are considered the preferred choice.

## 6. Conclusions and Future Perspectives

Obviously, the topographic orientation of scaffolds has a significant impact on both the biological characteristics of various cells and their specific tissue regeneration. For the majority of tissues, aligned scaffolds are the preferred alternative over random scaffolds for tissue repair and regeneration. However, it is worth noting that even within the same tissue, the optimal choice of scaffold topographic orientation may be different for different layers and cell types. For the repair of certain specific locations, a mixed topography of scaffolds may be most efficient. The underlying mechanisms of why scaffold topographic orientation modulates cellular behaviors are complex. The most widely accepted theory is that scaffold topographic orientation regulates cell morphology by “contact guidance”, and subsequently results in different cell morphologies. Although there have been a large number of studies on the effect of scaffold topographic orientation for tissue regeneration, most are limited to in vitro and in vivo animal experiments. Clinical trials, such as randomized controlled trials, are lacking. For the successful translation of tissue engineering technology in the broad clinical field, it is crucial that a large number of clinical trials are carried out in the future.

To fully understand the relationship between scaffold topographic orientation and cell behaviors, as well as to develop customized topographical scaffolds for certain tissue repair, several key issues need to be further investigated. Firstly, the more specific molecular mechanisms and the long-term interactions between topographical scaffolds and implanted tissue sites need to be further explored. Secondly, to better mimic the microenvironment in vivo, the difference between the 3D topographical structure and 2D topographical structure needs to be studied. Finally, future research should focus on improving the manufacturing processes to increase the production efficiency of tissue engineering scaffolds and to find more suitable biomaterials to enable large-scale tissue production for clinical use.

## Data Availability

Not applicable.
